# Long-term overall survival from a phase I trial using intratumoral plasmid interleukin-12 with electroporation in patients with melanoma

**DOI:** 10.1186/1479-5876-13-S1-O3

**Published:** 2015-01-15

**Authors:** Adil Daud, Kathryn Toshimi Takamura, Tu Diep, Richard Heller, Robert H Pierce

**Affiliations:** 1 University of California, San Francisco – San Francisco, California, USA; 2 OncoSec Medical Inc. – San Diego, California, USA; 3 Old Dominion University – Norfolk, Virginia, USA

## Background

In 2007, a phase 1 dose-escalation safety study of intratumoral electroporation (EP) of plasmid interleukin-12 (pIL-12) was completed in 24 patients (pts) with malignant melanoma. IL-12 is thought to promote interferon-γ-pathway activation and antigen presentation and processing machinery (APM) to enhance systemic anti-tumor response. This phase 1 study was the first ever gene therapy in humans to use EP to locally deliver DNA plasmid to induce systemic anti-tumor immunity. Here we present long-term overall survival (OS) results from this study.

## Methods

Twenty-four pts with stage IIIB-IV melanoma and with at least two accessible lesions received one treatment cycle of pIL-12 EP administered on days 1, 5 and 8. Dose escalation with increasing plasmid concentrations was performed in cohorts 1 through 5. Plasmid was dispensed at concentrations of 0.1, 0.25, 0.5, 1.0 and 1.6 mg/mL, and plasmid volume was calculated based on tumor volume. Two additional cohorts were enrolled (cohorts 6 and 7) and received a total dose of 3.8 or 5.8 mg/treatment respectively, divided among two to four tumor sites, irrespective of tumor volume. Tumor responses were evaluated by modified RECIST. Biopsies were obtained for tumor histology, including evaluation of lymphocytic infiltrate, and tissue intratumoral IL-12 concentration.

## Results

In this phase 1 study, 53% (10/19) pts with metastatic disease had a systemic response to the local pIL-12 EP treatment as evidenced by stable disease or objective regression of non-injected lesions. Additionally, 11% (2/19) experienced complete regression of all distant lesions without concurrent or subsequent systemic therapy. The most frequent adverse event (AE) related to treatment was transient pain during the EP procedure, with 54% (13/24) pts reporting grade 1 and 46% (11/24) pts reporting grade 2 pain. No DLT was noted within any of the treatment cohorts. The median OS (n=24, ITT) was 24.2 mos. In pts who experienced SD or better with pIL-12 EP (n=9), OS of 46.4 mos was observed, a 32.9 mos increase compared to non-responders (OS 13.5 mos; n=15).

## Conclusions

Results from this phase 1 study demonstrate that local treatment with pIL-12 EP successfully induces systemic anti-tumor immune-mediated effects without severe local or systemic toxicity. Moreover, long-term follow-up data suggest that systemic disease stabilization with pIL-12 EP is correlated with improved survival. Based on this evidence, intratumoral EP of pIL-12 is an effective tool for gene transfer of DNA plasmid with potential applications as both a monotherapy and in combination with other agents that promote anti-tumor immunity.

